# Photobiomodulation therapy’s impact on angiogenesis and osteogenesis in orthodontic tooth movement: in vitro and in vivo study

**DOI:** 10.1186/s12903-023-03824-z

**Published:** 2024-01-31

**Authors:** Jietong Zhong, Xinyu Zhang, Yaru Ruan, Yue Huang

**Affiliations:** 1 School of Stomatology, Southwest Medical University, Sichuang, Luzhou, China; 2 The Second People’s Hospital of Yibin, Yibin, Sichuang China; 3 School of Stomatology, Jinan University, Guangzhou, Guangdong China

**Keywords:** Low-level laser therapy, Orthodontic treatment, Co-culture, Bone formation, Angiogenesis

## Abstract

**Background:**

This study explores the effectiveness of Photobiomodulation Therapy (PBMT) in enhancing orthodontic tooth movement (OTM), osteogenesis, and angiogenesis through a comprehensive series of in vitro and in vivo investigations. The in vitro experiments involved co-culturing MC3T3-E1 and HUVEC cells to assess PBMT’s impact on cell proliferation, osteogenesis, angiogenesis, and associated gene expression. Simultaneously, an in vivo experiment utilized an OTM rat model subjected to laser irradiation at specific energy densities.

**Methods:**

In vitro experiments involved co-culturing MC3T3-E1 and HUVEC cells treated with PBMT, enabling a comprehensive assessment of cell proliferation, osteogenesis, angiogenesis, and gene expression. In vivo, an OTM rat model was subjected to laser irradiation at specified energy densities. Statistical analyses were performed to evaluate the significance of observed differences.

**Results:**

The results revealed a significant increase in blood vessel formation and new bone generation within the PBMT-treated group compared to the control group. In vitro, PBMT demonstrated positive effects on cell proliferation, osteogenesis, angiogenesis, and gene expression in the co-culture model. In vivo, laser irradiation at specific energy densities significantly enhanced OTM, angiogenesis, and osteogenesis.

**Conclusions:**

This study highlights the substantial potential of PBMT in improving post-orthodontic bone quality. The observed enhancements in angiogenesis and osteogenesis suggest a pivotal role for PBMT in optimizing treatment outcomes in orthodontic practices. The findings position PBMT as a promising therapeutic intervention that could be seamlessly integrated into orthodontic protocols, offering a novel dimension to enhance overall treatment efficacy. Beyond the laboratory, these results suggest practical significance for PBMT in clinical scenarios, emphasizing its potential to contribute to the advancement of orthodontic treatments. Further exploration of PBMT in orthodontic practices is warranted to unlock its full therapeutic potential.

**Supplementary Information:**

The online version contains supplementary material available at 10.1186/s12903-023-03824-z.

## Background

Orthodontics is a field dedicated to applying specific orthodontic forces to teeth, thereby altering the periodontal microenvironment, inducing bone remodeling and regeneration. This ultimately leads to changes in tooth position and partial reconstruction of the alveolar bone, resulting in improved teeth alignment and facial aesthetics. The quality of osteogenesis post-movement is crucial for achieving successful clinical outcomes. Orthodontic tooth movement (OTM) involves the simultaneous processes of alveolar bone resorption on the pressure side and new bone formation on the tension side [1, 2]. Osteoclasts play a pivotal role in bone resorption, while osteoblasts contribute to bone regeneration Additionally, the process of tooth movement is influenced by changes in periodontal microcirculation, emphasizing the importance of local microvessel formation and nutrient supply through microvessels for tissue regeneration and repair [3, 4].

Bone neoformation and vascular neoformation are intertwined processes that significantly impact osteogenesis. Blood vessels play a vital role in sustaining cell viability by ensuring perfusion within the healing zone during physiological development or bone regeneration [5]. Vessel elongation and cellular compaction initiate the metabolism of osteogenic stress proteins. The gradual transition back to aerobic conditions, facilitated by neoangiogenesis, ensures the enduring nature of the newly formed osseous structures [6]. Vascular endothelial growth factor-A (VEGF-A) is a key player in angiogenesis, stimulating endothelial cell migration, proliferation, and influencing both osteogenic and angiogenic processes [5]. VEGF expression is necessary for approximately 4 weeks to establish enduring blood vessels. The balance between VEGF-induced angiogenesis and osteogenesis requires meticulous exploration through a controlled release approach of VEGF at a low concentration, employing a long-term sustained delivery system [7, 8]. Within the complex periodontal microcirculation, vascular endothelial cells (ECs) interact with bone cells like osteoblasts, mesenchymal stromal cells, and osteoclasts, leading to the formation of microcapillary networks around newly formed bone tissue [9]. These blood vessels facilitate nutrient and oxygen delivery and provide pathways for various cells to access the growing bone. This neovascularization is critical for tissue repair and organ development, especially in the context of bone formation [10, 11].

In the periodontal microenvironment, an intricate interplay unfolds among cell types like human periodontal ligament cells (HPDLCs), vascular endothelial cells, osteoblasts, and osteoclasts. This dynamic interaction is mediated by various signaling molecules and pathways, resulting in osteogenic-angiogenic coupling [11, 12]. Notably, two primary subtypes of vascular endothelial cells, H-type and L-type, are involved in osteogenesis, with H-type vessels playing a dominant role [13, 14].

Research has highlighted the significance of Notch signaling in H-type vascular endothelial cells, which mediates osteogenic-angiogenic coupling. Noggin protein, controlled by the NOG gene, enhances osteoblast and chondrocyte activity, contributing to bone formation [12]. This intricate network of signaling pathways underscores the close connection between osteogenesis and angiogenesis.

Photobiomodulation therapy (PBMT), defined as a form of light therapy that uses non-ionizing light sources, including lasers, light-emitting diodes and/or broadband light, in the visible (400–700 nm) and near-infrared (700–1100 nm) electromagnetic spectrumhas [15] been shown to expedite OTM and reduce patient discomfort [16]. However, specific clinical guidelines for PBMT dosage and frequency in accelerated OTM are lacking. In the context of bone enhancement, PBMT has been found to promote cellular proliferation and osteogenic differentiation in vitro and induce a state of hypermetabolism in bone remodeling [16–18]. Diode lasers with specific energy densities and wavelengths have shown promise in enhancing cell proliferation and migration, even under hypoxic conditions. Mechanistically, PBMT enhances cellular metabolism by providing energy to mitochondria, ultimately promoting bone remodeling [19–21]. Molecular investigations into PBMT’s effects have demonstrated increased expression of genes and proteins associated with osteogenesis and angiogenesis. However, the full efficacy of PBMT in promoting osteoblast proliferation, differentiation, and bone regeneration during OTM requires further investigation [22].

Regarding vascularization, PBMT has shown potential in reducing pro-inflammatory and pro-coagulant activities in vascular endothelial cells, which could support angiogenesis [23–25]. Nevertheless, the role of PBMT in promoting vascular endothelial cell proliferation and angiogenesis during OTM remains unclear.

Given these uncertainties, further research is needed to elucidate the biological effects and mechanisms of lasers in fostering angiogenesis and new bone formation during OTM. This study employs a 980 nm diode laser to establish an in vitro co-culture system involving MC3T3-E1 and HUVEC cells and conducts in vivo animal experiments to investigate the impact of PBMT on osteogenesis and angiogenesis in rats undergoing OTM within the periodontal microcirculation. This comprehensive approach aims to shed light on the effects of PBMT on osteoblasts and vascular endothelial cells during the OTM process in rats.

## Methods

### Cell culture

MC3T3-E1 and HUVEC cells (KeyGEN BioTECH, Nanjing, China) in good condition were chosen and co-cultured at ratios of 10:1 (MC3T3-E1: HUVEC) decided by pre-experiment in α-MEM containing 10% FBS and 1% penicillin/streptomycin (Oricell, Guangzhou, China) at 37 °C with 5% CO2.

### Cell proliferation assay

This experiment employed a super-fine fiber diode laser manufactured by Intensive Medical & Dental System Private Limited, with a wavelength of 980 nm, a laser power of 0.5 W, and a spot area of approximately 0.2826cm^2^. This configuration yielded an energy density of 1.7 J/cm^2^ per unit of time. The total energy within a unit of time was measured using a specialized energy density meter, and the energy attenuation, when compared to the laser’s theoretical output, fell within an acceptable range. Consequently, energy density was controlled for each experimental group based on varying irradiation times. The cell proliferation experiment was designed with six distinct laser parameter groups, corresponding to irradiation times of 0 s, 1 s, 2 s, 3 s, 4 s, and 5 s, resulting in energy densities of 0 J/cm^2^, 1.7 J/cm^2^, 3.5 J/cm^2^, 5.3 J/cm^2^, 7.1 J/cm^2^, and 8.8 J/cm^2^, respectively. Cell viability and proliferation in the co-culture system were assessed using the Cell Counting Kit-8 (CCK-8, Dojindo, Tokyo, Japan) at 12 h, 24 h, 48 h, and 72 h following stimulation with various laser energy densities. MC3T3-E1 and HUVEC cells were co-seeded at a 1:1 ratio (2 × 10^3 cells/well) in 96-well plates, with four replicate wells for each group, along with an additional blank control group. Laser irradiation was administered to designated wells in accordance with the experimental design after 12 hours of incubation, utilizing varying irradiation durations corresponding to different energy densities. Subsequent to laser irradiation, the well plates were further incubated for 12 h, 24 h, 48 h, and 72 h. CCK-8 reagent was subsequently added to each well, and the optical density (OD) value at 450 nm was measured using a microplate reader (Bio-rad, iMark™ Microplate Absorbance Reader, California, USA) following a 2-hour incubation.

### In vitro experimental grouping and laser irradiation protocol

The experiment comprised four groups: MC3T3-E1 group, HUVEC group, MC3T3-E1 + HUVEC group, and MC3T3-E1 + HUVEC+PBMT group. In the co-culture groups for alkaline phosphatase detection experiments, alizarin red staining, and q-PCR experiments, MC3T3-E1 cells were mixed with HUVEC cells at a 10:1 cell ratio. For the tube formation experiment, MC3T3-E1 and HUVEC cells were mixed at a 1:1 cell ratio. The experimental group received daily cell irradiation with an energy density of 5.3 J/cm^2^.

### ALP activity assay

Cells from each group were seeded in 24-well plates at a density of 2 × 10^4 cells per well, maintaining consistent culture conditions and cell densities. Upon reaching 80–90% confluence, each group underwent osteogenic induction using MC3T3-E1 cell osteogenic differentiation induction medium (Oricell, Guangzhou, China) for a duration of 7 days. Subsequently, ALP staining was performed using the BCIP/NBT kit (Beyotime Institute of Biotechnology, Shanghai, China), and Alkaline Phosphatase activity was assessed using the Alkaline Phosphatase Activity Assay Kit (Biosharp, Hefei, China).

### Alizarin red stain

Cells from each group were seeded in 24-well plates at a density of 2 × 10^4 cells per well, maintaining consistent culture conditions and cell densities. When the cell confluence reached 80–90%, cells were cultured with MC3T3-E1 cell osteogenic differentiation induction medium for a period of 14 days. The experimental group received daily irradiation with an energy density of 5.3 J/cm^2^. After 14 days, all groups were stained with Alizarin Red (Beyotime Institute of Biotechnology, Shanghai, China).

### Tube formation test

Tube formation tests were used to detect the ability to generate vessels. In each well of a 48-well plate, 200 μL of Matrigel was carefully added, ensuring no bubbles, and allowed to solidify. MC3T3-E1 and HUVEC cells were seeded at 4 × 10^4 cells per well on the solidified Matrigel (Corning, NY, USA) coated plate in groups: MC3T3-E1, HUVEC, MC3T3-E1 + HUVEC, and MC3T3-E1 + HUVEC+PBMT. The latter group received a single 5.3 J/cm^2^ laser irradiation at the beginning. After 6 hours of incubation, capillary-like structures were observed under an inverted microscope, and the degree of tube formation was analyzed by counting the number of complete tubes.

### Quantitative real-time PCR

Cells from each group were seeded in 24-well plates at a density of 2 × 10^4 cells per well, maintaining consistent culture conditions and cell densities. When cell confluence reached 80–90%, cells were cultured with MC3T3-E1 cell osteogenic induction medium for 7 days. Subsequently, cells in the well plates were harvested, and total RNA was extracted using the Total RNA Extraction Kit (Novozymes Biotechnology, Nanjing, China). The extracted total RNA was quantified using a UV spectrophotometer, and only samples with a 260:280 ratio > 1.8 were utilized for subsequent experiments. cDNA was synthesized with a reverse transcription kit (Novozymes Biotechnology, Nanjing, China), and this cDNA was employed to assess the expression of bone formation-related markers (RUNX2, COL-1α1), angiogenesis markers (VEGF, HIF-1α), and key osteogenic markers like osteocalcin (OCN). This analysis was carried out using the Polycomb monochrome fluorescent quantitative PCR system (RT-PCR) and the Bio-Rad myiQ monochrome fluorescence quantitative PCR system, with normalization using the glyceraldehyde 3-phosphate dehydrogenase (GAPDH). The gene-specific primers were commercially synthesized (Sangon, Shanghai, China), and their sequences are listed in Table 1. The acquisition and analysis of qPCR data were conducted employing the QuantStudio™ 3 Real-Time PCR System (Thermo Scientific). Relative expression levels were determined using the 2^-ΔΔCt^ method, with normalization carried out using the Ct value of the reference gene GAPDH.

**Table 1:** List of primer sequences used in Real-time PCR

Gene	Primer sequence
GAPDH(human)	Forward:CAAGGCTGTGGGCAAGGTCATC Reverse:GTGTCGCTGTTGAAGTCAGAGGAG
GAPDH(mouse)	Forward:TGAAGGTCGGTGTGAACGGATTTG Reverse:TCGCTCCTGGAAGATGGTGATGG
VEGF	Forward:GCCTTGCCTTGCTGCTCTACC Reverse:CTTCGTGATGATTCTGCCCTCCTC
HIF-1α	Forward:ACCGCTGAAACGCCAAAG Reverse:TCCATCGGAAGGACTAGGTGTCT
COL-1α1	Forward:GACAGGCGAACAAGGTGACAGAG Reverse:CAGGAGAACCAGGAGAACCAGGAG
OCN	Forward:CAAGCAGGAGGGCAATAAGGTAGTG Reverse:CGGTCTTCAAGCCATACTGGTCTG
RUNX2	Forward:GCAGCACTCCATATCTCTACT Reverse:TTCCGTCAGCGTCAACAC

### The orthodontic tooth movement model

Twenty-eight male Sprague-Dawley (SD) rats, 6 weeks old, were randomly assigned to one of the following experimental groups: (1) Control group (C group, *n* = 4), (2) Orthodontic Tooth Movement (OTM) without Photobiomodulation Therapy (PBMT) group (OTM group, *n* = 4), (3) OTM with PBMT at energy densities of 1.7 J/cm^2^ group (1.7 J/cm^2^ group, *n* = 4), (4) OTM with PBMT at energy densities of 3.5 J/cm^2^ group (3.5 J/cm^2^ group, *n* = 4), (5) OTM with PBMT at energy densities of 5.3 J/cm^2^ group (5.3 J/cm^2^ group, *n* = 4), (6) OTM with PBMT at energy densities of 7.1 J/cm^2^ group (7.1 J/cm^2^ group, *n* = 4), and (7) OTM with PBMT at energy densities of 8.8 J/cm^2^ group (8.8 J/cm^2^ group, *n* = 4). General anesthesia was administered to each rat through intraperitoneal injection of 3% pentobarbital sodium according to the ethical guidelines. A 0.30 mm stainless-steel ligature wire was then passed through the palatal adjacent space between the left first and second molar teeth to create a channel. Subsequently, a 0.20 mm orthodontic stainless steel ligature wire was passed through the channel and secured against the cervical portion of the first molar using a stainless steel spring. A dynamometer was employed to ensure a spring tension of 50 g. Another 0.20 mm ligature wire was passed through the spring and secured at the proximal incisor end of the spring. The distal mesial and palatal sides of the maxillary incisors were ligated using a specialized tool to create a 0.5-mm-deep retention groove, and the ligature wire was tightly secured to the incisors, ensuring alignment with the marked tension position. The sharp end of the ligature wire was covered with fluid resin. Penicillin (800,000 U, 0.5 mL/rat) was intramuscularly administered to each rat immediately after the operation and on postoperative days 2 and 3. One rat experienced a fatality due to an overdose of anesthesia during the procedure, while the remaining rats exhibited no abnormalities. After a 14-day period, the rats were euthanized, and tissue samples were collected for analysis. All procedures were approved by the Laboratory Animal Welfare and Ethics Committee of Jinan University (approval number: IIACUC-20221109-02).

### In vivo experimental animal laser irradiation protocol

The rats in the PBMT groups received laser irradiation based on their respective subgroups’ energy density by positioning a probe against the left maxillary first molar. Irradiation was performed at four specific points: the proximal and distal areas in the middle of the palatal side of the first molar, and the proximal and distal areas in the middle of the buccal side of the first molar. This irradiation procedure was conducted once every 24 hours. Rats were anesthetized daily through intraperitoneal injection of 3% sodium pentobarbital. Following the onset of anesthesia, the stability of the force device was assessed, and any signs of inflammation or damage to the gingival and mucosal tissues were examined. If dislodgement occurred, immediate reattachment was performed. Laser irradiation was then administered following the prescribed irradiation protocol. The body weights of the rats were monitored every 3 days throughout the 14-day irradiation period.

### Orthodontic tooth movement distance measurement

After 14 days of orthodontic tooth movement, the rats were euthanized with an intraperitoneal overdose of 3% sodium pentobarbital. The maxillary bone was carefully extracted from each rat using tissue scissors, cleaned to remove excess tissues, and the distance from the cervical part of the first molar to the cervical part of the second molar on the left side of the maxilla was measured using vernier calipers. This measurement was performed for each rat in groups 0 to 5. All measurements were conducted in triplicate by three different operators, and the results were subsequently averaged.

### Tissue staining

The maxillae from the rats, collected 14 days after orthodontic tooth movement, were initially fixed in 4% paraformaldehyde for a duration of 24 hours. Subsequently, the specimens were immersed in a 10% EDTA decalcification solution for the decalcification process. The decalcification solution was replaced on a weekly basis, and the complete decalcification process spanned approximately 4 weeks. Following successful decalcification, continuous tissue sections with a thickness of 5 μm were prepared using the traditional paraffin embedding method. These sections were oriented in the horizontal direction. Hematoxylin and eosin staining were employed for the purpose of observing neovascularization and new bone tissue. Immunohistochemical staining was performed by incubating the sections overnight at 4 °C with primary antibodies, followed by treatment with horseradish peroxidase-conjugated goat anti-rabbit streptavidin secondary antibodies at room temperature for 2 hours. Subsequently, the sections were stained with hematoxylin staining solution and sealed for subsequent observation.

### Statistical analysis

One-way and two-way analyses of variance (ANOVA) were performed to detect the significant differences, followed by Tukey’s test as a post hoc comparison. Statistical analyses were performed.

## Results

### Effect of PBMT on viability and proliferation of the MC3T3-E1 and HUVEC co-culture system

**Fig. 1:**
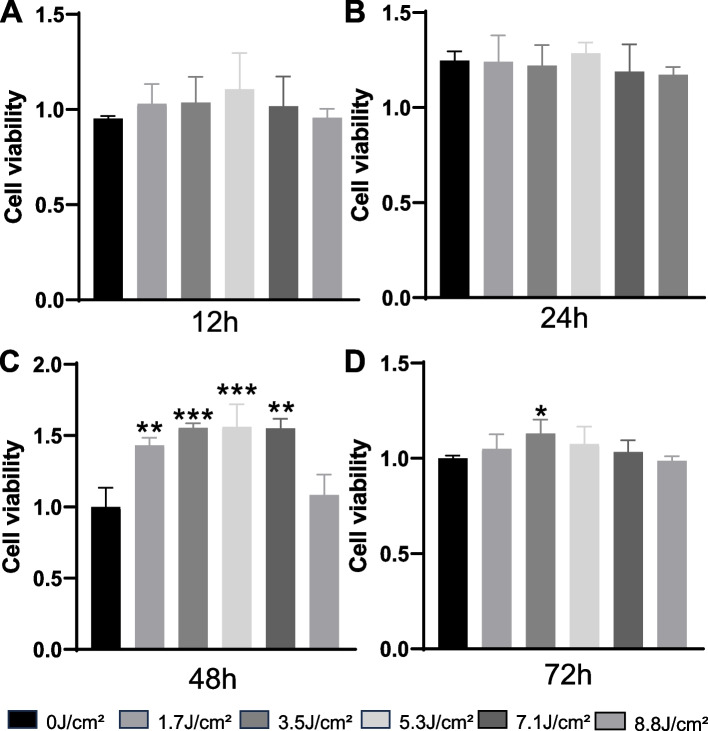
Impact of Varied Energy Density of the laser on Co-Culture Systems Over Different Time Durations. **A** Co-culture system with varying energy density levels for 12 hours. **B** Co-culture system with varying energy density levels for 24 hours. **C **Co-culture system with varying energy density levels for 48 hours. **D** Co-culture system with varying energy density levels for 72 hours. (**: *P* < 0.01, ***: *P* < 0.001; compared to the control group)

The viability and proliferation of the MC3T3-E1 and HUVEC co-culture system under PBMT were assessed using the CCK-8 assay. Statistical analysis was performed using one-way analysis of variance to compare each energy irradiation group with the control group. The results (Fig. 1) indicated that when cells in the co-culture system were irradiated with laser at different energy densities and assessed at 12 hours and 72 hours, the cell viability in the experimental groups initially increased and then decreased. Notably, at 12 hours, 5.3 J/cm^2^, and at 72 hours, 3.5 J/cm^2^, promoted cell proliferation in the co-culture system. However, there were no statistically significant differences in cell viability between the experimental groups and the control group. At 24 hours, there were no statistically significant differences in cell viability between the experimental groups and the control group. However, at 48 hours, energy densities of 1.7 J/cm^2^, 3.5 J/cm^2^, 5.3 J/cm^2^, and 7.1 J/cm^2^ significantly promoted cell proliferation in the co-culture system, with statistically significant differences (*P* < 0.05). In contrast, an energy density of 8.8 J/cm^2^ showed no significant promoting effect compared to the control group. These results suggest that PBMT can enhance cell proliferation in the co-culture system, with 5.3 J/cm^2^ at 48 hours showing statistically significant improvements compared to the control group, indicating it may be the optimal energy density for promoting co-culture system proliferation.

### Impact of PBMT on osteogenic potential in the co-culture system of MC3T3-E1 and HUVEC cells

**Fig. 2:**
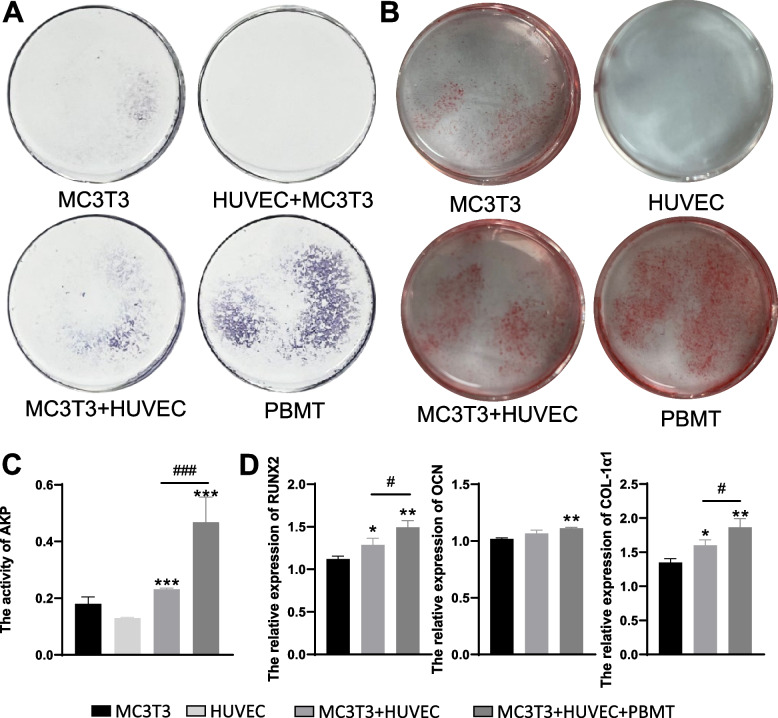
Impact of Photobiomodulation Therapy (PBMT) on Osteogenic Differentiation in Co-Culture Systems. **A** ALP staining for assessing osteogenic differentiation. **B** Evaluation of osteogenic differentiation through Alizarin Red S staining. **C** Alkaline Phosphatase Activity Assay results (**: *P* < 0.001, compared to the MC3T3-E1 group; ###: *P* < 0.001, compared to the MC3T3 + HUVEC group). **D** Measurement of Runx2, OCN, and COL-1α1 expression levels by qRT-PCR (: *P* < 0.05, **: *P* < 0.01; compared to the M group; #: *P* < 0.05; compared to the MH group)

After 7 days of osteogenic induction, the ALP staining results (Fig. 2A) exhibited bluish-purple staining in all groups except the HUVEC group. Notably, in the co-culture group with PBMT intervention, the bluish-purple positive staining area was larger than that in the MC3T3-E1, HUVEC, and co-culture groups without PBMT intervention. Additionally, the co-culture group without PBMT showed a larger positive staining area than the MC3T3-E1 monoculture group. These findings suggest that co-culture may enhance the osteogenic differentiation of bone cells, and PBMT, when applied in conjunction with co-culture, further promotes osteogenic differentiation within the co-culture system.

We conducted Alizarin Red staining on cells after 14 days of osteogenic induction (Fig. 2B). Except for the HUVEC group, the other three groups displayed visible red-stained calcification nodules. Remarkably, the co-culture group exhibited a larger positive staining area compared to the MC3T3-E1 group alone, and the PBMT-treated co-culture group displayed a larger positive staining area than both the MC3T3-E1 group and the MC3T3-E1 and HUVEC co-culture group. This outcome implies that co-culturing may enhance the mineralization capacity of cells, and PBMT may promote osteogenic mineralization within the co-culture system.

Quantitative analysis of intracellular alkaline phosphatase (AKP) levels (Fig. 2C) revealed that the co-culture group with PBMT intervention exhibited higher AKP expression compared to the MC3T3-E1, HUVEC, and co-culture groups without PBMT intervention. Statistically significant differences were observed between the MC3T3-E1 monoculture group and the co-culture group with PBMT intervention (*P* < 0.05). These findings suggest that the combined effect of co-culture and PBMT may result in improved osteogenic differentiation.

qRT-PCR results (Fig. 2D) demonstrated that the co-culture of MC3T3-E1 and HUVEC cells led to higher relative expression levels of the osteogenesis-related genes RUNX2, OCN, and COL-1α1 compared to the MC3T3-E1 group and the HUVEC group when cultured individually. Furthermore, the relative expression of RUNX2 and COL-1α1 exhibited statistically significant differences between the MC3T3-E1 group and the standard co-culture group. Additionally, in the PBMT-intervened co-culture group, the relative expression levels of RUNX2 and COL-1α1 were also higher than in the standard co-culture group, and this difference was statistically significant (*P* < 0.05). However, there was no statistically significant difference in OCN expression between the MC3T3-E1 group and the co-culture group, and PBMT intervention in the co-culture group also did not yield statistically significant differences compared to the standard co-culture group (*P* > 0.05). These results suggest that co-culturing enhances the osteogenic differentiation capacity of osteoblasts and indicate that PBMT can augment the osteogenic potential within the co-culture system.

### The influence of PBMT on angiogenesis in co-culture systems

**Fig. 3:**
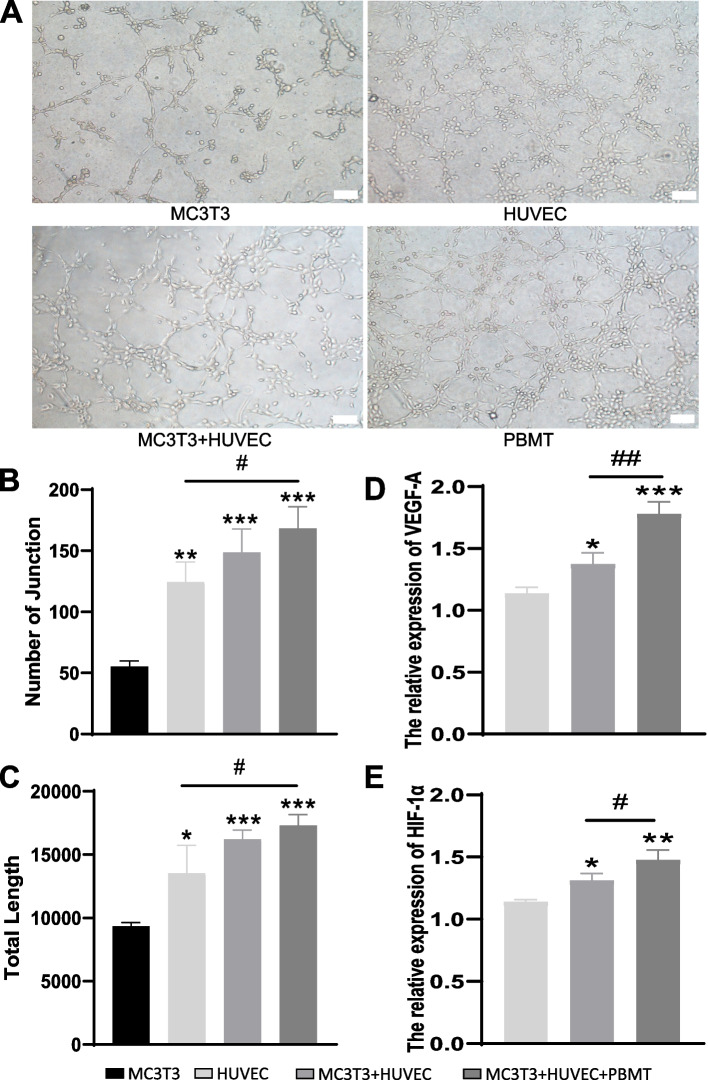
The Impact of PBMT on Angiogenesis in Co-Culture Systems. **A** Representative images of tube formation in MC3T3-E1, HUVEC, and MC3T3-E1/HUVEC co-culture systems under light microscopy (100x magnification, scale bars represent 100 μm). **B** Quantification of the number of tubular branches in each experimental group (**: *P* < 0.01, **: *P* < 0.001; compared to the MC3T3-E1 group; #: *P* < 0.05; compared to the HUVEC group). **C** Statistical analysis of the total length of tubular branches in each experimental group (: *P* < 0.05, **: *P* < 0.001; compared to the MC3T3-E1 group; #: P < 0.05; compared to the HUVEC group). **D**-**E** Measurement of HIF-1α and VEGF expression levels by qRT-PCR (: *P* < 0.05, **: *P* < 0.01, ***: *P* < 0.001; compared to the HUVEC group; #: *P* < 0.05, ##: *P* < 0.01; compared to the MC3T3-E1 + HUVEC group)

The Tube Formation Test results (Fig. 3A) demonstrated the presence of tube-like structures in all groups. Quantitative analysis (Fig. 3B, C) revealed that the co-culture group displayed a higher number of branch points and a greater total branch length compared to the monoculture groups of both cell types. Furthermore, the co-culture group with PBMT intervention exhibited even more branch points and a greater total branch length than the co-culture group without PBMT, with statistically significant differences observed, particularly in the case of HUVEC with PBMT intervention (*P* < 0.05). These findings suggest that while co-culture alone may have a limited effect on promoting angiogenesis, the addition of PBMT treatment to the co-culture system significantly enhances its angiogenic potential. The qRT-PCR results (Fig. 3D-E) revealed that the relative expression of vascular-related genes HIF-1α and VEGF in the co-culture group of MC3T3-E1 and HUVEC cells was higher than in the MC3T3-E1 group and the HUVEC group when cultured individually. Furthermore, the relative expression of HIF-1α and VEGF exhibited statistically significant differences between the HUVEC group and the standard co-culture group. Additionally, in the PBMT-intervened co-culture group, the relative expression levels of HIF-1α and VEGF were also higher than in the standard co-culture group, and this difference was statistically significant (*P* < 0.05). These findings suggest that co-culturing enhances the expression of angiogenic differentiation capacity in endothelial cells, and PBMT enhances the angiogenic potential within the co-culture system.

### Measurement of tooth movement distance in different groups of rats

**Fig. 4:**
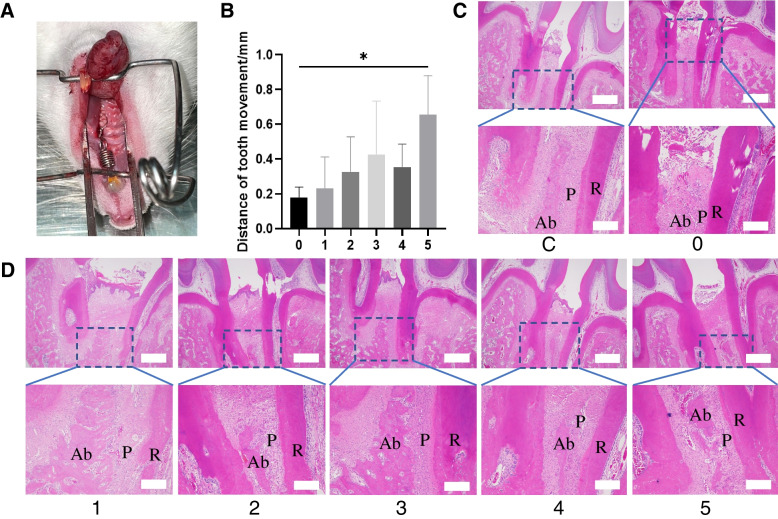
Animal Model and H&E Staining. C represents the control group, while 0–5 correspond to various levels of laser radiation characterized by energy densities of 0 J/cm^2^, 1.7 J/cm^2^, 3.5 J/cm^2^, 5.3 J/cm^2^, 7.1 J/cm^2^, and 8.8 J/cm^2^. **A** Establishment of the Orthodontic Tooth Movement Rat Model. (**B**) Statistical Analysis of Mesial Movement Distance of the Left Maxillary First Molar in Each Group (*: *P* < 0.05, compared to the OTM group). **C**-**D** Hematoxylin and Eosin (**H**&**E**) stained sections (40×, upper row, scale bar = 500 μm; 100×, lower row, scale bar = 200 μm). P denotes the Periodontal Ligament; Ab signifies Alveolar Bone; R represents Tooth Root

Following the application of orthodontic forces in the mesial direction to the left maxillary first molar (Fig. 4A)of rats for a duration of 14 days, we collected maxillary bone specimens for analysis. Utilizing a vernier caliper, we meticulously measured the shortest distance between the mesial aspect of the first molar and the distal aspect of the second molar. The statistical analysis of tooth movement distances for each experimental group is illustrated in Fig. 4B. Our results indicate that rats subjected to laser treatment at various energy densities exhibited significantly greater movement of the left maxillary first molar in the mesial direction when compared to the group without laser treatment (Group OTM), with Group 8.8 J/cm^2^ demonstrating particularly noteworthy differences (*P* < 0.05). These findings strongly suggest that PBMT has the potential to enhance orthodontic tooth movement. Notably, when comparing tooth movement distances among the experimental groups, Group 8.8 J/cm^2^ exhibited greater tooth movement distances compared to the control group. (*P* > 0.05).

### Results of tissue staining

Observations from Hematoxylin and Eosin (HE)-stained tissue sections (Fig. 4C and D) revealed that in the control group of rats, the periodontal ligaments on both sides of the maxillary first molars exhibited nearly uniform width, regularly arranged collagen fibers, and an intact internal structure, indicative of a normal physiological state. In contrast, all experimental groups displayed highly active metabolism within the periodontal tissues after 14 days of orthodontic force application. The periodontal ligament on the tension side of the maxillary first molars exhibited relative widening due to stretching, while on the pressure side, it showed narrowing due to compression. Upon examination of tissue remodeling within the periodontal ligament on the tension side of the maxillary first molars in all groups, we observed dilated blood vessels and new bone deposition along the distribution of collagen fibers in the stretched periodontal ligament. Moreover, compared to the control group, all experimental groups exhibited a significant increase in newly formed blood vessels. Notably, a substantial number of osteoblasts were observed protruding onto the surface of the tooth roots on the tension side, indicative of active osteogenic activity.

**Fig. 5:**
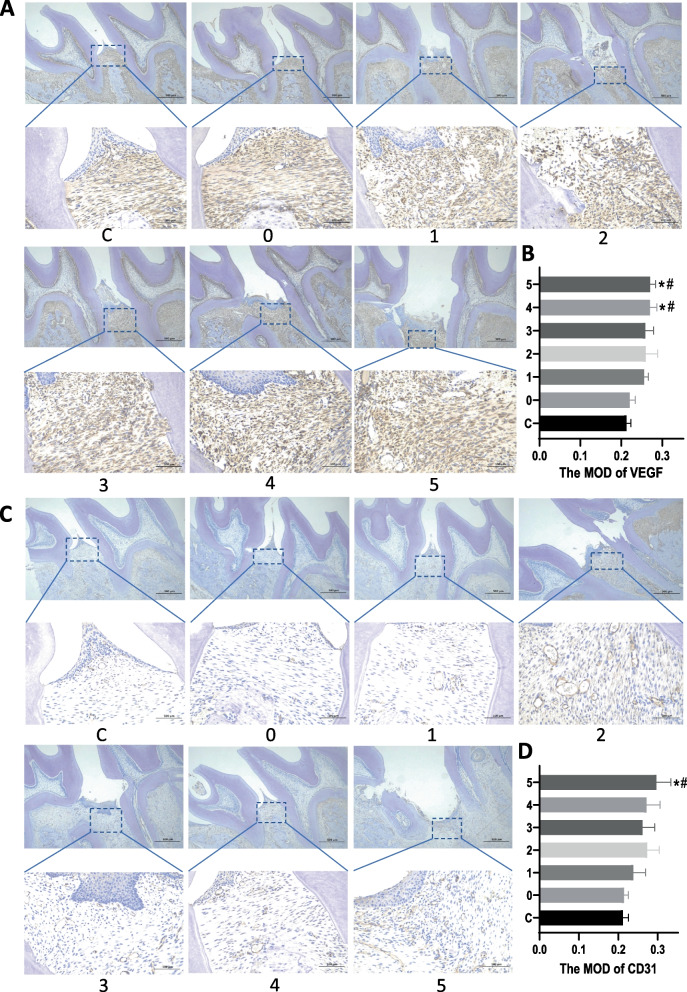
Immunohistochemical Analysis of VEGF and CD31 Expression. C represents the control group, while 0–5 correspond to various levels of laser radiation characterized by energy densities of 0 J/cm^2^, 1.7 J/cm^2^, 3.5 J/cm^2^, 5.3 J/cm^2^, 7.1 J/cm^2^, and 8.8 J/cm^2^. **A** Immunohistochemical staining results of VEGF in various groups, showing OTM lateral maxilla and first molar sagittal tissue sections at 40x magnification, as well as local periodontal tissue of the distal root of the first molar at 200x magnification. **B** Comparison of VEGF expression in the periodontal ligament on the tension side between the control group and the experimental group(*: *P* < 0.05; compared to the control group; #: *P* < 0.05,; compared to the OTM group). **C** Immunohistochemical staining results of CD31 in different groups, presenting OTM lateral maxilla and first molar sagittal tissue sections at 40x magnification, and local periodontal tissue of the distal root of the first molar at 200x magnification. **D** Comparison of the expression levels of CD31 in the periodontal ligament on the tension side between the control group and the experimental group(*: *P* < 0.05; compared to the control group; #: *P* < 0.05,; compared to the OTM group)

Immunohistochemical staining was performed on the upper 1/3 of the periodontal ligament area on the mesial aspect of the left maxillary first molar root of rats. The results (Fig. 5A, C) revealed varying degrees of brownish-yellow precipitates, with brownish-yellow cytoplasm and blue-stained nuclei, representing positive expression of Vascular Endothelial Growth Factor (VEGF) and CD31. Quantitative analysis demonstrated increased VEGF and CD31 (Fig. 5B, D) expression in the laser treatment group compared to the control group. Statistically significant differences were observed, with Group 8.8 J/cm2 exhibiting a more pronounced effect on promoting angiogenesis during orthodontic tooth movement. These findings suggest that PBMT has the potential to enhance vascularization, with specific energy densities, particularly 8.8 J/cm2, showing a more significant impact on promoting angiogenesis during orthodontic tooth movement.

## Discussion

Numerous investigations have demonstrated the favorable photobiomodulatory effects of PBMT across a spectrum of cellular processes, including cell proliferation, endothelial cell angiogenesis, osteogenic differentiation, bone regeneration, vascularization, and fracture healing. Currently, there is a considerable amount of research on PBMT, with most applications utilizing wavelengths ranging from 600 to 1100 nm, output powers ranging from 1 to 500 mW, and energy densities ranging from 0.1 to 100 J/cm2. In this experiment, a 980 nm wavelength diode laser was employed, which is consistent with the laser wavelength used in the study conducted by Wang [26]. Their study results indicated that LLLT within the range of 2 to 6 J/cm^2 can promote the proliferation and osteogenesis of PDLSCs, with 6 J/cm^2 being the most effective energy density. However, at an energy density of 8 J/cm^2, a noticeable inhibition of osteogenic differentiation in hPDLSCs was observed. This is similar to the cell proliferation experiment results in this study. The optimal energy density determined in this experiment for PBMT application was 5.3 J/cm^2. Interestingly, at 24 hours and 72 hours, the energy density of 8.8 J/cm^2 demonstrated an inhibitory effect on cell proliferation. However, several studies in the past have indicated significant variations in PBMT parameters for orthodontic tooth movement (OTM). Differences in parameters such as wavelength, energy density, power density, spot area, irradiation time, and number of irradiations can lead to diverse experimental outcomes [27]. PBMT has been observed to enhance the proliferative and osteogenic capacities of human periodontal ligament stem cells (hPDLSCs), and it influences cellular functions by activating cytochrome C oxidase and stimulating intracellular mitochondrial metabolic activities and ATP production [28, 29]. In terms of bone formation, two principal pathways are recognized: intramembranous osteogenesis and endochondral osteogenesis. Both pathways underscore the interactive nature of bone generation and vascularization, wherein osteogenesis arises from the dynamic interplay between vascular endothelial cells and osteoblasts. Prior research has substantiated that the co-culture of endothelial cells and mesenchymal stem cells (MSCs) can stimulate the osteogenic differentiation of stem cells [30]. Furthermore, PBMT treatment augments their osteogenic potential. MC3T3-E1 cells represent osteogenic precursor cells capable of differentiating into osteoblasts, thereby possessing the inherent characteristics of osteoblasts. Consequently, the co-cultivation of human umbilical vein endothelial cells (HUVEC) with MC3T3-E1 cells theoretically holds the potential to potentiate the osteogenic differentiation of MC3T3-E1 cells.

In the conducted experiment, results from ALP staining at day 7 of osteogenic induction in PBMT-treated cells suggested a greater extent of blue-purple expression in the PBMT-treated co-culture group compared to the other groups. This observation indicated that PBMT might have enhanced osteogenic differentiation within the co-culture system. Statistical analysis of AKP quantitative assay results further supported this observation, revealing a significant distinction between the MC3T3-E1 group and the co-culture group under PBMT intervention. This implied that the combined presence of PBMT and co-culture facilitated cell osteogenic differentiation. Furthermore, alizarin red staining on the 14th day of osteogenic induction and PBMT-treated cells demonstrated a significant enhancement in mineralization capacity within the PBMT-treated co-culture group relative to the other groups.

RUNX2 is a facilitator of early osteoblast differentiation [28], while COL-1α contributes significantly to the extracellular matrix of osteoblasts, playing a pivotal role in both bone mineralization and matrix formation [31]. OCN serves as a marker for osteoblast maturation, effectively reflecting osteoblast activity and bone transformation levels [32]. These factors closely correlate with osteoblast differentiation. HIF-1α is a crucial isoform of hypoxia-inducible factor-1 (HIF-1), a transcription factor governing cellular response to oxygen levels. HIF-1α regulates numerous factors related to angiogenesis and vasculogenesis [3, 30]. Additionally, VEGF is a pivotal mediator of endothelial cell proliferation and differentiation, significantly contributing to angiogenesis [33]. The qRT-PCR results from this experiment showed that PBMT augmented the relative expression of RUNX2, COL-1α1, HIF-1α, and VEGF within the HUVEC and MC3T3-E1 co-culture system. The tube-forming assay results further demonstrated that co-cultivation, coupled with PBMT, amplified the tube-forming capability of HUVEC compared to HUVEC cultured in isolation. This finding indicated that PBMT effectively promoted the vascularization potential of vascular endothelial cells.

These results align with the work of Bai [20], who established a co-culture system of bone marrow-derived mesenchymal stem cells (BMSCs) and HUVEC. Their study demonstrated that co-culture enhanced the osteogenic and angiogenic differentiation of cells compared to separate BMSC and HUVEC cultures. The combination of co-culture and PBMT further augmented these differentiation capacities. These findings underscore that co-culture fosters cellular communication and enhancement of proliferation and differentiation through interaction, while the application of PBMT serves to amplify these effects by influencing cell metabolism. Notably, the feedback loop involving reactive oxygen species (ROS) and HIF-1α also contributes to the regulation of angiogenesis through PBMT. By adjusting oxygen metabolism, PBMT sustains HIF-1α levels, subsequently elevating VEGF secretion and promoting endothelial cell proliferation and angiogenesis.

Moreover, potential mechanisms of osteogenesis and angiogenesis under hypoxia may involve the ERK pathway. Research indicates that ERK-mediated osteogenic differentiation can be enhanced under hypoxic conditions [34]. In a co-culture scenario involving PDLSCs and vascular endothelial cells, osteogenic differentiation can be further augmented via pathways such as COX2/PGE2/VEGF. Activation of ERK has also been linked to angiogenesis [35, 36]. Additional signaling pathways implicated in the osteogenesis and angiogenesis coordination between vascular endothelial cells and osteoblasts encompass the Notch and Wnt signaling pathways. Research suggests that the Notch signaling pathway regulates angiogenesis and bone formation between vascular endothelial cells and osteoblasts [37]. Activation of the endogenous Wnt signaling pathway and BMP signaling by HUVEC could potentially influence osteogenic differentiation in human BMSCs [38]. These pathways represent areas for further exploration in subsequent experimental studies to comprehend the mechanisms underlying PBMT-mediated osteogenic-angiogenic interactions between MC3T3-E1 and HUVEC. The research also aims to elucidate how PBMT impacts interactions between vascular endothelial cells and osteoblasts within the periapical microcirculation during OTM, including potential effects on vascular-related factor secretion and angiogenesis via these signaling pathways, thereby promoting bone neoformation.

Orthodontic tooth movement (OTM) involves a dual process encompassing bone resorption and bone remodeling neogenesis. On the pressure side subjected to compression, active osteoclasts contribute to alveolar bone resorption, while on the tension side experiencing stretching, bone remodeling becomes prominent with heightened osteoblastic activity, leading to increased bone formation. The findings from this experimental study indicate that the laser-irradiated rat groups exhibited more aggregated osteoblasts along the periodontal membrane-alveolar bone junction, accompanied by a greater deposition of new bone on the alveolar bone surface on the tension side. This contrasted with the periodontal tissues of the OTM group without laser irradiation.

VEGF plays a significant role in regulating both physiological and pathological angiogenic processes, affecting vascular permeability and maintenance [39]. Elevated VEGF secretion enhances vascular endothelial cell proliferation, migration, and differentiation, thereby promoting neovascularization. Within periodontal tissues, VEGF expression is observed in vascular endothelial cells, osteoblasts, and fibroblasts [40]. CD31, on the other hand, is primarily expressed in vascular endothelial cells. Given that VEGF governs vascular neogenesis, which subsequently promotes bone neogenesis, it is apparent that VEGF serves as a pivotal element in the bone formation process. As CD31 represents a marker of vascular endothelial cells, angiogenesis can be inferred through the examination of VEGF and CD31 expression levels [41].

Mechanical force during OTM potentially activates the P13k/Akt/mTOR signaling pathway, which in turn influences osteoblasts and vascular smooth muscle cells. Additionally, PBMT demonstrates a positive impact on flap vascularization. A study involving PBMT irradiation of BMSCs and mouse dorsal skin flap illustrated that the combination of PBMT and BMSCs enhances flap survival and neovascularization [25]. The present study’s results reveal substantial VEGF expression in rat periodontal tissues during OTM, suggesting that orthodontic force triggers active VEGF secretion from various cells in the periodontal tissues, with a more robust positive expression observed in the laser-irradiated group compared to the non-laser irradiation group. This indicates that orthodontic force induces blood vessel vasodilation within periodontal membranes, augmenting VEGF secretion and promoting angiogenesis within the periodontal tissue. PBMT, in turn, can further amplify vascularization in the periodontal tissue during OTM. Furthermore, it has been noted that the increased presence of osteoblasts on the tension side of rat periodontal tissues and the elevated VEGF secretion post-orthodontic force align with the current experiment’s outcomes. Tanya utilized PBMT during OTM and the subsequent retention stage, observing enhanced bone redeposition following orthodontic treatment by increasing bone formation and diminishing osteoclast redistribution on the pressure side of the OTM process [42].

As earlier discussed, vascularization significantly influences bone remodeling during OTM, with promoting vascularization and bone neoformation representing pivotal factors in enhancing osteogenesis quality following orthodontic treatment. Improved osteogenesis quality subsequently reduces the risk of orthodontic relapse. However, research on orthodontic relapse and the application of PBMT to OTM for enhancing osteogenic quality and mitigating orthodontic relapse remains limited. The current experiment investigated PBMT’s effects on osteogenesis and angiogenesis through an in vitro co-culture system and in vivo animal experiments during OTM in rats. The findings suggest that PBMT can potentially promote osteogenic and vascular differentiation, along with angiogenesis during OTM. Notably, the variation in energy absorption rates of laser between cells and living organisms underscores the distinction between cell and animal experiments. This discrepancy may contribute to the different optimal energy densities required for these contexts. Further research is warranted to elucidate potential discrepancies in mechanisms, inviting additional investigations to be conducted in the future. Moreover, while the current study provides a phenomenological understanding of PBMT’s impact on osteogenesis and angiogenesis in periodontal tissues, extensive experimental research is essential to unravel the underlying mechanisms behind these effects.

## Conclusion

This study aimed to investigate the effects of Low-Level Laser Therapy (PBMT) on osteogenesis and angiogenesis during the process of Orthodontic Tooth Movement (OTM). A co-culture system comprising MC3T3-E1 and HUVEC cells was established in vitro to explore the impact of PBMT on osteogenic and angiogenic differentiation within the co-culture system. Osteogenic induction and the application of PBMT were used to assess and compare the osteogenic capacity of MC3T3-E1 cells in co-culture systems with and without PBMT treatment. The angiogenic ability of HUVEC cells was evaluated through tube formation assays, and qRT-PCR experiments were conducted to measure the relative expression levels of osteogenic and angiogenic-related genes in MC3T3-E1, HUVEC, and the co-culture system in response to PBMT.

Furthermore, an in vivo experiment using rats with orthodontic tooth movement (OTM) models was conducted to investigate the influence of PBMT on osteogenesis and angiogenesis within periodontal tissues. The following conclusions were drawn:

PBMT at an energy density of 5.3 J/cm^2^ significantly promoted the in vitro proliferation of MC3T3-E1 and HUVEC cells within the co-culture system, enhancing their osteogenic and angiogenic differentiation capabilities. This was associated with an upregulation of osteogenic-related genes, including RUNX2 and COL-1α, as well as angiogenic-related genes, such as HIF-1α and VEGF.

During the process of OTM in rats, vascular formation primarily occurred within the tension-side periodontal membrane, where numerous dilated blood vessels were observed. The PBMT-treated group exhibited higher levels of positive expression of VEGF and CD31, along with a greater accumulation of osteoblast cells and increased deposition of new bone on the tension-side alveolar bone surface.

In future research, we plan to conduct more comprehensive experiments to delve deeper into the mechanisms through which PBMT regulates osteogenesis and angiogenesis during the OTM process. This will provide a theoretical foundation for potential clinical applications in the future.

### Supplementary Information

**Additional file 1.**

## Data Availability

All data generated or analysed during this study are included in this published article and its supplementary information files.

## Abbreviations

ALP/AKP: alkaline phosphatase
α-MEM: α-modified eagle’s medium
BMPs: bone morphogenetic proteins
BCA: butyleyanoacrylate
CD31: platelet endothelial cell adhesion molecule-1
CGF: concentrate growth factors
COL-1α1: type I collage
CM: complete medium
CCK-8: cell counting kit-8
cDNA: complementary DNA
COX2: Cyclooxygenase
DMSO: dimethyl sulfoxide
EMCN: endomucin
ECs: endothelial cells
EDTA: ethylenediaminetetraacetic acid
ERK: Extra cellular signal-related kinase
FBS: fetal bovine serum
g-DNA: genomic DNA
GAPDH: Glyceraldehyde-3-phosphate dehydrogenase
PBMT: Photobiomodulation Therapy
HPDLCs: human periodontal ligament cells
hPDLSCs: human periodontal ligament stem cells
HUVEC: human umbilical vein endothelial cells
HE: hematoxylin-eosin
HRP: horseradish peroxidase
HIF-1α: hypoxia inducible factor-1α
