# Supplementary material for: Photobiomodulation therapy’s impact on angiogenesis and osteogenesis in orthodontic tooth movement: in vitro and in vivo study
Source: BMC Oral Health. 2024 Jan 31;24:147. doi: 10.1186/s12903-023-03824-z (PMC10832110; doi:10.1186/s12903-023-03824-z)
Supplement: Supplementary file 1 — Additional file 1. [file 12903_2023_3824_MOESM1_ESM.docx]

CCK8

12H

24H

48H

72H

Tube formation test

Cd31

| control | 0 | 1 | 2 | 3 | 4 | 5 |
| --- | --- | --- | --- | --- | --- | --- |
| 0.221848 | 0.225141 | 0.229117 | 0.30214 | 0.244962 | 0.231586 | 0.334917 |
| 0.21732 | 0.202833 | 0.272166 | 0.276148 | 0.296863 | 0.282046 | 0.292366 |
| 0.194738 | 0.21345 | 0.211395 | 0.240273 | 0.242004 | 0.299395 | 0.260363 |

VEGF

| control | 0 | 1 | 2 | 3 | 4 | 5 |
| --- | --- | --- | --- | --- | --- | --- |
| 0.200346 | 0.208082 | 0.266914 | 0.292223 | 0.276963 | 0.25588 | 0.282708 |
| 0.214423 | 0.235088 | 0.253537 | 0.235452 | 0.236855 | 0.289277 | 0.271538 |
| 0.222499 | 0.217483 | 0.245379 | 0.25089 | 0.261282 | 0.263232 | 0.256826 |

QPCR

RUNX2

OCN

COL-1α1

HIF-1α

VEGF-A

Distance

0.00 0.18

0.00 0.24

0.00 0.12

1.70 0.12

1.70 0.14

1.70 0.44

3.50 0.20

3.50 0.22

3.50 0.56

5.30 0.22

5.30 0.28

5.30 0.78

7.10 0.20

7.10 0.42

7.10 0.44

8.80 0.98

8.80 0.46

8.80 0.56

8.80 0.62
